# Anesthetic management for laryngeal closure: retrospective evaluation of 50 cases

**DOI:** 10.1186/s40981-017-0140-3

**Published:** 2017-12-22

**Authors:** Midori Mogami, Chiaki Nemoto, Makoto Kano, Mariko Muto, Youichi Tanaka, Mashahiro Murakawa

**Affiliations:** 1 Department of Anesthesiology, Ohara General Hospital, 6-11 Ohomachi, Fukushima, 960-8611 Japan; 2 Department of Otorhinolaryngology, Head and Neck, Ohara General Hospital, 6-11 Ohmachi, Fukushima, 960-8611 Japan; 3 Department of Anesthesiology, Fukushima Medical University School of Medicine, 1 Hikarigaoka, Fukushima, 960-1295 Japan

**Keywords:** Laryngeal closure, Aspiration pneumonia, Malnutrition

## Background

Laryngeal closure is effective in patients who suffer from repeated aspiration pneumonia [1]. Most patients who require this surgery, however, present with malnutrition or are relatively dehydrated either because of aspiration pneumonia or their primary disease. We report our anesthetic management protocol and discuss the problems encountered in these patients.

## Case presentation

After obtaining approval from the hospital’s ethics committee, we evaluated the medical records of patients who underwent surgical closure of the larynx in our hospital from January 2013 to January 2015. The patients comprised 35 men and 15 women with a mean body mass index of 18 ± 3.9 kg/m^2^ and mean age of 71 ± 18.4 years. Most patients had malnutrition, with a serum albumin level of 2.7 ± 0.7 g/dl and serum sodium level of 136.0 ± 5.4 mEq/l. Their primary diseases varied as shown in Table 1. In total, 17 (34%) of the patients had already undergone tracheostomy, and oral tracheal intubation was performed in two others. Two patients required mechanical ventilation. Preoperative blood gas analysis showed a PaO_2_ of 88.6 ± 23.9 mmHg and PaCO_2_ of 41.7 ± 5.7 mmHg.

**Table 1:** Patients’ primary diseases

	Total numbers	Post-tracheotomy	Intubated
Parkinson’s disease	10	2	1
Cerebral hemorrhage	9	5	
Cerebral infarction	9	5	
Cerebral palsy	5		1
Multiple system atrophy	2	2	
Amyotrophic lateral sclerosis	2	1	
Dementia	3		
Disuse syndrome	10	2	

Disuse syndrome: postoperative dissecting aneurysm (*n* = 1), postoperative major abdominal surgery (*n* = 3), hypoxic brain damage due to suffocation (*n* = 1), pneumonia (*n* = 5)

The operation time was 158.4 ± 24.6 min, and the duration of anesthesia was 202.5 ± 27.1 min. The anesthetic induction agents varied among the patients: sevoflurane was used in 36 patients, midazolam in 7, propofol in 5, and thiopental in 1. In most patients, general anesthesia was maintained by sevoflurane and remifentanil. Target-controlled infusion by propofol and remifentanil was performed in only one patient, who had amyotrophic lateral sclerosis. Rocuronium was administered in all patients. In 30 patients, rocuronium was administered only at the time of tracheal intubation. Additional doses of rocuronium were needed in 20 patients. The mean induction dose of rocuronium was 0.79 ± 0.16 mg/kg, with additional doses of 0.42 ± 0.34 mg/kg as needed. During anesthesia, 38 patients required a cardiovascular agent. Among them, 11 (22%) were given ephedrine and 33 (66%) were given phenylephrine. Continuous administration of noradrenaline was performed in two patients and dopamine hydrochloride in one patient. Forty-two patients (84%) required reversal of rocuronium using sugammadex sodium, and eight patients (16%) were given naloxone because of delayed emergence of spontaneous breathing. With the exception of two patients who were ventilated before the operation, none of the patients required mechanical ventilation during the postoperative period. Postoperative pain management with acetaminophen or non-steroidal anti-inflammatory drugs was satisfactory.

## Discussion

Laryngeal closure lessens the risk of aspiration, making it possible for the patients to improve their nutritional condition. Malnutrition and/or hyponatremia are serious perioperative complications for these patients. Malnutrition is a possible factor associated with anastomotic leakage [2]. No patients who underwent laryngeal closure under general anesthesia in our hospital required re-operation due to anastomotic leakage.

Some of the patients had hyponatremia, which is sometimes found in elderly patients [3]. The mean preoperative sodium concentration was 136 ± 5.4 mEq/l in our patients, although five (10%) patients had a sodium level of <130 mEq/l. Radical perioperative fluid therapy could raise the sodium concentration to a point that causes osmotic demyelination syndrome [4]. We administered 1% glucose lactated Ringer’s solution (2.9 ± 1.1 ml/kg/h) during anesthesia in our patients. Surgical closure of the larynx poses little risk of massive bleeding. However, these patients were chronically dehydrated, so fluid administration was increased during the perioperative period.

During anesthesia, some of the patients have experienced hypotension. Malnutrition and chronically dehydrated state were possible factors of hypotension especially in elder patients. In most cases, we could manage by intermittent administration of ephedrine and phenylephrine.

In the operating rooms, emergence from anesthesia might be delayed by remifentanil; however, no patients showed respiratory suppression in the recovery room. In some patients who need this operation, the primary disease causes a consciousness disorder, which was present before anesthesia. Therefore, the use of drugs that could affect their respiratory condition or state of consciousness should be carefully considered. Although the effect of remifentanil is short, even in patients with renal or hepatic failure [5], reversal drugs might be needed because of delayed emergence from anesthesia. None of our patients developed a worsened respiratory condition postoperatively.

## Conclusions

Patients who require laryngeal closure present with several preoperative problems due to repeated aspiration pneumonia or their primary disease. Despite these problems, no serious complications associated with general anesthesia were found in our hospital.
